# Four Cases of Glossitis

**Published:** 1853-04

**Authors:** T. D. Strong

**Affiliations:** Westfield, N. Y.


ART. III. — *Four Cases of Glossitis.* By T. D. STRONG, M. D., Westfield, N. Y.

The infrequency of Glossitis, its severity and danger, as well as my personal appreciation of the intense suffering incident to the disease, prompt me to report the following four cases, with the results of treatment:

CASE I. Thomas Doherty, Irish, aged 22, laborer.

*Sept.* 18*th*, 1851. He used some bad tobacco, and tongue commenced swelling.

20*th*. First saw him this P. M. Tongue nearly double its natural size, swelling mostly confined to one side of raphe. Red and glossy. Complains of great pain in it. Saliva flowing freely. Articulation indistinct. No general febrile movement.

℞. Sulph. magnes. et sennæ, freely.

21*st*, 9, A. M. Rather more swollen and painful. Scarified it superficially, but only little blood flowed. No fever.

℞. Hyd. sub. mur. grs. v, at four hours.

8, P. M. Much more swollen and painful, and heavy yellow coat upon it. Cannot articulate at all. Respiration somewhat obstructed. Skin hot. Pulse 96, and bounding.

℞. Venesection ℥xx.

and soon followed by four large Swedish leeches under tongue. Sulph. magnes.

Leeches drew well, and the bites bled freely. He expressed himself "better," and could articulate indistinctly.

Resolution went on rapidly during night, and in the morning he sent me word that he was almost well. Two days after cam eto office with scarcely a trace of the disease remaining.

CASE II. This was one of personal interest, being myself.

*Oct.* 10*th and* 11*th*. Felt some soreness of fauces, with swelling of left tonsil, and a slight aphthous exudation. Gargled with infusion of capsicum.

*12th*, morn. Tongue commenced swelling 1½ inches from apex, mostly confined to one side. Soreness of fauces subsided.

℞. Sulph. mag.

It advanced through the day, felt rigid, and became painful. Could swallow fluids only.

*13th*. Dr. Henn called. Did not advise active antiphlogistic treatment. Feared gangrene.

℞. Gargle of chlorid. sod.

Grew worse. At eve applied four leeches under tongue with temporary relief to pain.

*14th*. During latter part of night had grown rapidly worse. Could not talk a word. Saliva flowed profusely. Pain intense, a deep throbbing, and a superficial, like inserting hot needles. System but little affected. Pulse 80, and soft. No heat of skin. Drs. Henn and Spencer called. Dr. S. bled me ℥xvj, without much effect. Disease advanced till the

*17th*. Tongue protruded. Teeth more imbedded in it. Was covered with a most thick dense yellow coat.

2, P. M. At my request Dr. H. gave me chloroform and laid freely open. Some pus and considerable blood escaped. Pain was entirely relieved, and in a few minutes I slept the sleep of the weary. Convalescence proceeded rapidly, and in five days the incision had healed.

Some induration remained at seat of abscess.

Case III. Continuation of Case II.

In February, the induration increased slowly for a week, then inflammation of tongue commenced. Its course was more rapid, attended with less pain and swelling, and in three days fluctuation was evident. Opened abscess, and it discharged pus for several days. Took hydriod. pot. for some weeks, and induration entirely disappeared in March. No indications of a return to this time.

Case IV. Patrick O'Connell. Irish laborer. Aged 30.

*Oct. 30th*, 1852. Attack commenced the 29th, could assign no cause.

*30th*, 2, P. M. Tongue somewhat swollen, most at root, and advancing toward apex, both sides alike. Smooth and shining. Increased flow of saliva. No constitutional disturbance. Sublingual muscles involved.

℞. Hyd. sub. mur., grs. v,

P. Doveri, at 4 hours, give 3 times, and follow by sulph. mag.,

Gargle. Vinegar, ℨiv,

Mur. ammon., ℨij,

Tr. opii, ℨj.

31*st*, 9, A. M. Swelling increased rapidly. Tense, shining appearance, except in center, where thick yellow coat is collecting. Tongue protrudes. Cannot articulate at all. Pulse 100, full and hard. Skin hot.

℞. Venesection ℥xxx to syncope,

Sulph. mag., ℥iss. and irritation externally to throat.

*Nov.* 1*st.* Resolution rapidly taking place. Talks indistinctly. Pulse 80, and soft. Tongue cleaning.

℞. Light diet.

Discharged him next day.

From my own observation, and the reported cases within my reach, I believe too much reliance is placed on the "expectant" treatment in this disease. Its course is too rapid, and an unfavorable result too certain, to trust to palliatives, or the "vis medicatrix naturæ." During the five months that the induration remained in my tongue, my mind was constantly haunted with fears of fresh attacks, or (what is more to be dreaded) of malignant disease, and an occasional twinging pain at the seat of induration was not well suited to allay those fears. The determination then became fixed that no one under my care should ever suffer the same, either in mind or body, if active measures would produce resolution and avoid abscess. In neither case did I have reason to regret an *over activity*, but in my own, I regretted, with tears, my "masterly *inactivity*" in the early stage.

In Case I, the effect of the leeches, following venesection, was so satisfactory, that I relied upon them in my own case, but a previous venesection was lacking to prevent reaction. In Case IV I had no leeches, and so was more severe in the general treatment.

In the Journal of April, 1852, page 651, is a reference to Cases I and II, and I was surprised at both the facts and inferences drawn from the partial notes given in my letter. In my own case the symptoms were not "highly aggravated." The next day no trace remained of three of the bites. The other one was inflamed, but it was the one farthest from the most highly inflamed part of the tongue, and that inflammation subsided very soon, before the glossitis reached its climax, so I did not attribute the final result to any bad effects of leech bites.

The leeches were applied on the 13th, and the "free incision" was not made till the 17th, after pus had formed. In Case I, *no* "free incision" was made. My notes say that I "scarified it superficially, and but little blood flowed," and it had no effect in arresting the disease, as shown by the farther history of the case.

*February* 26, 1853.

